# Disability and functioning assessment of women with RSI/WRMSDs: the use of the ICF checklist

**DOI:** 10.5327/Z1679443520190443

**Published:** 2019-12-01

**Authors:** Francesca de Brito Magalhães, Mônica Angelim Gomes de Lima, Robson da Fonseca Neves, Katia Costa-Black, Tânia Maria de Araújo, Lauro Antonio Porto

**Affiliations:** 1 Graduate Program in Health, Environment and Work, School of Medicine, Universidade Federal da Bahia - Salvador (BA), Brazil. Universidade Federal da Bahia Graduate Program in Health, Environment and Work School of Medicine Universidade Federal da Bahia Brazil; 2 Graduate Program in Physical Therapy, Universidade Federal da Paraíba - João Pessoa (PB), Brazil. Universidade Federal da Paraíba Graduate Program in Physical Therapy Universidade Federal da Paraíba Brazil; 3 Occupational and Industrial Orthopaedic Center, NYU Langone Health - New York (NY), United States. Occupational and Industrial Orthopaedic Center NYU Langone Health United; 4 British Standard Institutions Environmental Health and Safety Services - Hillsboro (OR), United States. British Standard Institutions Environmental Health and Safety Services United States; 5 Health Department, Universidade Estadual de Feira de Santana - Feira de Santana (BA), Brazil. Universidade Estadual de Feira de Santana Health Department Universidade Estadual de Feira de Santana Brazil

**Keywords:** occupational health, return to work, International Classification of Functioning, Disability and Health, rehabilitation

## Abstract

**Background:**

Comprehensive approaches using the International Classification of Functioning, Disability and Health (ICF) Checklist have been adopted to give more visibility to demands related to specific health situations.

**Objective:**

To analyze the incapacity and functioning associated with activity/participation and environmental factors of female workers with repetitive strain injury/work-related musculoskeletal disorders (RSI/WRMSDs) using workers’ narratives, ICF codes and the RSI/WRMSDs Checklist developed as a part of the present research project.

**Methods:**

A qualitative analysis of in-depth interviews with 15 female workers diagnosed with RSI/WRMSDs was completed. Functioning and disability were assessed by linking ICF codes identified in the participants’ narratives to those included in the RSI/WRMSDs Checklist.

**Results:**

Fifty-three of 60 ICF codes included in the RSI/WRMSDs Checklist were detected in the participants’ narratives. Related to activity/participation, 26 codes were identified and 27 related to environmental factors.

**Conclusion:**

These results highlight the significance of the RSI/WMSDs Checklist to detect clinical and social problems experienced by workers during the rehabilitation and return-to-work process. They also reinforce the relevance of expanding the application of the checklist to male and female workers with RSI/WRMSDs undergoing rehabilitation to attain other levels of validation.

## RESUMO |

**Introdução:**

As abordagens compreensivas utilizando o modelo do *checklist* da Classificação Internacional de Funcionalidade, Incapacidade e Saúde (CIF) vêm sendo utilizadas para dar visibilidade às demandas em situações de saúde específicas.

**Objetivo:**

Avaliar a incapacidade e funcionalidade relacionadas à atividade/participação e a fatores ambientais apresentadas por trabalhadoras com lesões por esforço repetitivo/distúrbios osteomusculares relacionados ao trabalho (LER/DORT) por meio da associação entre suas narrativas, os códigos da CIF e o do checklist LER/DORT criado por profissionais.

**Métodos:**

Foi realizado um estudo qualitativo com 15 trabalhadoras com diagnóstico de LER/DORT que responderam a uma entrevista em profundidade. A avaliação da incapacidade e funcionalidade deu-se pela ligação entre os códigos da CIF identificados nas entrevistas e os que constavam do *checklist*.

**Resultados:**

Cinquenta e três dos 60 códigos da CIF presentes no *checklist* para LER/DORT foram identificados nas entrevistas. Acerca da atividade/participação, foram reconhecidos 26 códigos, e dos fatores ambientais, foram identificados 27 códigos.

**Conclusões:**

Esses resultados apontam que o *checklist* LER/DORT é um importante instrumento cujo uso pode facilitar a identificação dos aspectos clínicos e sociais vivenciados pelos trabalhadores no seu processo de reabilitação e retorno ao trabalho. Os achados deste estudo reforçam a importância da ampliação da aplicação do *checklis t*em trabalhadores e trabalhadoras com ­LER/­DORT em processo de reabilitação para que alcancemos outros níveis de sua validação.

## INTRODUCTION

Physical, emotional and mental distress derived from repetitive strain injury (RSI)/work-related musculoskeletal disorders (WRMSDs) is associated with social and personal impairments among workers, with consequences for their activities and social life. These conditions - which affect workers worldwide, and more particularly those in emerging economies, such as Brazil - pose a serious public health problem as a result of high rates of temporary or permanent disability and impact workers’ health, their families, and the healthcare and social security systems^1^^,^^2^^,^^3^.

Although the Brazilian National Occupational Health Policy prioritizes the prevention and surveillance of musculoskeletal symptoms in the workplace, the number of workers with chronic musculoskeletal complaints likely to interfere with their occupational life is increasing^4^. This fact evidences the need to investigate the gaps in prevention, care and rehabilitation.

Some experiences in Brazil indicate that when provided multidisciplinary rehabilitation and adequate support, workers with RSI/WRMSDs may return to work^5^. As a response to the need for developing more effective return-to-work interventions, a pilot program to rehabilitate workers with RSI/WRMSDs was established in Bahia^6^. This program is based on the Sherbrook biopsychosocial, ecological and case management model^7^, with the addition of initiatives for prevention of long-term disability and sustainable return to work^8^^,^^9^.

The theoretical framework for the development of a RSI/WRMSDs Checklist converges with the concept of prolonged work disability following Loisel et al.^10^ case management model, which explains work disability as a multidimensional phenomenon largely caused by poor management and communication of the systems related to workers’ care and rehabilitation (i.e. health care, compensation, workplace and personal systems). A sensitive approach to this problem should consider coordinated interventions on the individual-workplace-stakeholders interface. Concepts such as prevention of prolonged disability and sustainable return to work^8^ have influenced occupational health actions in Brazil, e.g. those coordinated by the Workers’ Reference Center of Piracicaba and the Workplace Surveillance and Health Care Department/Workers’ Reference Center of the State of Bahia, as concerns both their theoretical framework and effective implementation^5^^,^^6^.

The choice of parameters to assess functioning and disability among individuals with RSI/WRMSDs to design treatment and rehabilitation programs poses a challenge in terms of return to work and remaining at work. Assessment strategies which combine practical consensuses and scientific evidence are essential to implement valid and feasible actions along the rehabilitation process^9^^,^^11^. The International Classification of Functioning, Disability and Health (ICF) provides a scientifically sound and consensual classification system that comprises multiple dimensions relevant for the assessment of functioning and disability in different contexts and for different health conditions^1^. While it has been acknowledged as a practical instrument for international use, the practical application has proven to be difficult due to the large number of codes^1^. To facilitate its use, the World Health Organization suggests the development of a checklist using a shorter version of all the ICF codes^12^. While this *Checklist* does not have universal validity, one may argue that it affords realistic representations of sociopolitical environments, and thus represents a more pragmatic approach^12^^,^^13^^,^^14^^,^^15^^,^^16^. The result of this initiative was the ICF RSI/WRMSDs core set, later renamed ­RSI/­WRMSDs *Checklist*^17^. It comprises ICF codes related to RSI/WRMSDs selected by successive consensuses among occupational health experts.

In line with the ICF framework, the conceptual definition of Vocational Rehabilitation (VR)^11^ has been adopted for the present study, that is: “VR is a multi-professional evidence-based approach that is provided in different settings, services, and activities to working age individuals with health-related impairments, limitations, or restrictions with work functioning, and whose primary aim is to optimize work participation.

The aim of the present study was to assess functioning and disability relative to ICF domains activity/participation and environmental factors among female workers with RSI/WRMSDs through analysis of the correlation between their narratives on the experience of illness and the ­RSI/­WRMSDs Checklist.

## METHODS

A descriptive-exploratory approach was chosen to understand the chronic illness process experienced by workers on sick leave or not. Following Cieza et al.^18^, a thematic content analysis based on ICF codes was conducted.

Fifteen female workers were recruited by a convenience sampling from three different institutions in Salvador, Bahia: the Workers’ Reference Center of the State of Bahia, linked to the National Occupational Health Care Network; the medical department of a public judicial institution; and the outpatient pain clinic of Professor Edgard Santos University Hospital Complex, Federal University of Bahia. Participants were female workers with confirmed diagnosis of RSI/WRMSDs undergoing rehabilitation in 2010 and with impaired work ability, even when not necessarily on sick leave^6^. RSI/WRMSDs are conditions with gender specificity and predominate among females^19^. Thus, the focus of this study exclusively on women was to obtain an homogeneous sample with similar development of ­RSI/­WRMSDs^17^. It should be observed that cases among males and gender differences in the prevalence of disease have been previously discussed^20^^,^^21^; however, it is beyond the scope of this study.

The selected participants had been diagnosed with RSI/WRMSDs involving the neck and upper limbs and attributed at least one of the following International Classification of Diseases (ICD) codes: G56, M53, M65, M75 and M77. Other characteristics of the sample are described in Chart 1.

**Chart 1.** Participants’ characteristics, Salvador, Bahia, Brazil, 2010 (n=15).

Age (years)	Occupation	Educational level	Years since onset of disease
23	Saleswoman	Complete secondary school	1
41	Judicial technical assistant	Complete higher education	5
49	Cashier	Incomplete secondary school	3
43	General services assistant	Complete secondary school	5
45	Cashier	Complete secondary school	12
56	Cashier	Complete secondary school	12
48	Cashier	Complete secondary school	18
50	Cashier	Incomplete secondary school	10
53	Cashier	Incomplete secondary school	14
51	Cashier	Complete secondary school	13
43	General services assistant	Complete secondary school	6
46	Cashier	Complete secondary school	14
46	Production assistant	Incomplete secondary school	7
51	Cashier	Complete secondary school	10
48	Bank employee	Complete secondary school	15

The RSI/WRMSDs Checklist was developed by a multiprofessional group of occupational health experts. Their underlying principle was to use the basic knowledge from the different professionals in the multidisciplinary team and their practical experience to identify the most significant demands affecting workers with WRMSDS involving the neck and upper limbs, using the ICF codes as a guide^17^. Following a consensus between the experts, the term “core set” was changed to “checklist” to indicate the practical and clinical nature of this protocol to assess the functioning of workers with ­RSI/­WRMSDs^6^.

The final Checklist includes the following functions and their corresponding codes:

- Body functions (b) represented by mental, sensory, pain and neuromusculoskeletal functions, as well as movement-related functions;
- Body structures (s) represented by the nervous system and movement structures;
- Activity and participation (d) represented by individual mobility, domestic life, interpersonal interactions and relationships, and the major aspects of life; and finally,
- Environmental factors (e) represented by support and relationship, attitudes and services, systems and policies.

The experts involved in the development of the Checklist call attention to the fact that the checklist has limitation to detect demands related to the workplace, because the ICF codes do not include a detailed description of these factors^6^^,^^17^.

For the present study, only two checklist’s domains were considered, namely, activity/participation (30 codes) and environmental factors (33 codes). They were selected because they include factors susceptible to interventions in the work environment, organization and process, and thus are likely to contribute to VR^6^. In the ICF, *activity* is defined as the execution of a task or action, and *participation* as the act of being involved in daily life activities. The *environmental factors* make up the physical, social and attitudinal environment in which people live and conduct their lives^1^.

Data collection was performed by health care workers previously trained to administer the ICF and with experience with in-depth interviews. Interviews were individually performed and followed a semi-structured script that included topics such as the illness process, relationship between work, disease and everyday life, and VR facilitators and barriers. The number of participants was established based on the saturation criterion, i.e. the point in which no additional ICF codes were further added^22^. The interviews were transcribed *verbatim* at a later time.

A thematic content analysis technique was used to interpret the data collected in the interviews based on the frequency of each category that appeared in the narratives by comparing the identified ICF themes with categories of the RSI/WRMSDs Checklist. Relevant sections of interviews were considered as the thematic recorded units^18^. Data analysis involved three steps. First, interviews were transcribed and read to obtain a general idea of the collected data. In the second step, the thematic recorded units were identified if they related to functioning and disability of the workers and if they were they were describing activity/participation and environmental factors. In the final step, the identified units were linked to related ICF codes.

In this final step, guidelines formulated by Cieza et al.^18^ and used by other authors^21^^,^^23^ were followed. These guidelines provide detailed orientation to researchers on how to link narrative sections to ICF codes with focus on the precision of the recorded units and the meaning of each ICF code, as well as the identification of recorded units that are not included in the ICF codes.

The RSI/WRMSDs Checklist codes were linked to the participants’ narratives through the thematic recorded units, which embodied the demands present in the narratives, then the frequency of the identified codes were registered. To ensure uniformity of the data analysis, we linked the narrative sections to the ICF second level codes as suggested^1^. The third level codes were re-classified in the second level latter. The ICF codes found during qualitative analysis and not included in the RSI/WRMSDs Checklist were described as additional codes.

Several strategies were utilized to ensure the reliability of the data. First, a training exercise using one of the interview verbatim was completed by two independent researchers, i.e. the researcher-interviewer and another researcher. This exercise’s goal was to verify if they similarly coded the associate disability and functioning aspects to the thematic units using a written protocol. Next, this procedure was repeated with all the interview transcripts. Finally, a randomly selected sample representing 15% of the verbatim analyzed and encoded was reviewed by a third researcher trained in the use of the ICF use and in its rules^18^. Interrater reliability was high (above 90%) and the very few discrepancies were resolved by consensus between the three researchers.

The study was approved by the research ethics committee of Bahian School of Medicine and Public Health, ruling no. 64/2009, in compliance with the Ministry of Health and National Health Council Resolution no. 466/2012.

## RESULTS

The qualitative analysis included more than 11 hours of taped interviews, which resulted in 1,061 concepts related to ICF. Of these, 454 concepts (42.2%) corresponded to the domain activity/participation and 607 (57.8%) to environmental factors. They were linked to 64 second level ICF codes.

Table 2 shows the frequency of functioning and disability aspects related to the domain activity/participation. The most relevant thematic unit within this domain was “remaining in a paid job” According to all participants, the actual or possible chance of work disability was the main theme discussed. Table 1 shows the environmental factors. The participants presented as the main obstacles to rehabilitation were the lack of services as well as the lack of employment systems and policies.

**Table 1.** Domain environmental factors code linking, Salvador, Bahia, Brazil, 2010 (n=454).

ICF codes	Code description	Frequency
e110	Products and technology for personal consumption	25
e115	Products and technology for personal use in daily living	11
e120	Products and technology for personal indoor and outdoor mobility and transportation	0
e135	Assistive products and technology for employment	5
e140	Products and technology for culture, recreation and sport	1
e165	Assets	1
e225	Climate	2
e255	Vibration	0
e310	Immediate family	55
e315	Extended family	23
e320	Friends	3
e325	Products and technology for culture, recreation and sport	28
e330	People in positions of authority	21
e340	Personal care providers and personal assistants	4
e355	Health professionals	8
e360	Other professionals	2
e410	Individual attitudes of immediate family members	11
e415	Individual attitudes of extended family members	4
e420	Individual attitudes of friends	1
e425	Individual attitudes of acquaintances, peers, colleagues, neighbors and community members	24
e430	Individual attitudes of people in positions of authority	61
e445	Individual attitudes of strangers	2
e450	Individual attitudes of health professionals	22
e455	Individual attitudes of other professionals	3
e460	Societal attitudes	2
e465	Social norms, practices and ideologies	0
e520	Open space planning services, systems and policies	2
e540	Transportation services, systems and policies	2
e555	Associations and organizational services, systems and policies	3
e570	Social security services, systems and policies	60
e575	General social support services, systems and policies	1
e580	Health services, systems and policies	89
e585	Education and training services, systems and policies	7
e590	Labor and employment services, systems and policies	124

ICF: International Classification of Functioning, Disability and Health.

**Table 2.** Domain activity/participation code linking, Salvador, Bahia, Brazil, 2010 (n=607).

ICF codes	Code description	Frequency
d160	Focusing attention	4
d170	Writing	6
d177	Making decisions	0
d210	Undertaking a single task	3
d220	Undertaking multiple tasks	28
d230	Carrying out daily routine	31
d240	Handling stress and other psychological demands	16
d415	Maintain a body position	17
d430	Lifting and carrying objects	23
d440	Fine hand use	47
d445	Hand and arm use	25
d450	Walking	2
d455	Moving around	12
d470	Using transportation	10
d475	Driving	2
d510	Washing oneself	3
d520	Caring for body parts	17
d540	Dressing	4
d550	Eating	0
d570	Looking after one’s health	3
d630	Preparing meals	12
d640	Doing housework	57
d650	Caring for household objects	1
d660	Assisting others	4
d710	Basic interpersonal interactions	7
d740	Formal relationships	5
d750	Informal social relationships	1
d760	Family relationships	0
d770	Intimate relationships	1
d825	Vocational training	0
d830	Higher education	2
d845	Acquiring, keeping and terminating a job	2
d850	Remunerative employment	80
d855	Non-remunerative employment	2
d910	Community life	4
d920	Recreation and leisure	20
d930	Religion and spirituality	3

ICF: International Classification of Functioning, Disability and Health.

Data saturation was obtained after the 11^th^ interview. The codes identified in the 11^th^ and 12^th^ interviews were identical to those detected in the previous ones. The number of new codes found in the 13^th^ to 15^th^ interviews was insignificant, with only 3 new codes.

### ICF CODES AND THE RSI/WRMSDS CHECKLIST

**Figure 1.**
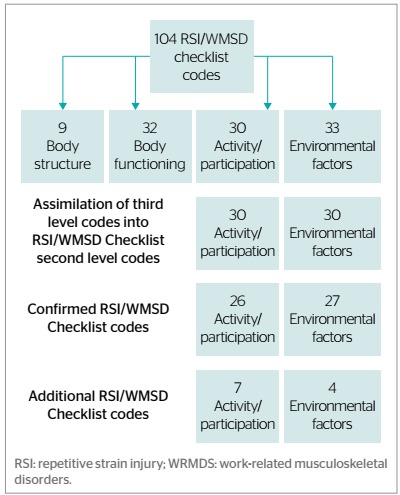
Flowchart of checklist code checking, Salvador, Bahia, Brazil, 2010 (n=64).

Fifty-three of the 60 ICF codes included in the ­RSI/­WRMSDs Checklist domains activity/participation and environmental factors were confirmed (Figure 1). Each of these codes were found more than 20 times in the participants’ narratives.

### ADDITIONAL CATEGORIES

Eleven ICF codes were also identified, which were not included in the RSI/WRMSDs Checklist: 7 relative to the domain activity/participation and 4 to environmental factors. Only 7 RSI/WRMSDs Checklist codes were not mentioned in the interviews (d177, d550, d760, d825, e120, e255 and e465).

## DISCUSSION

The overall characteristics of the sample were similar to those of active workers analyzed in studies of prevalence of RSI/WRMSDS, particularly in relation to their age. In 60% of such studies participants experience RSI/WRMSDs between the age of 30 to 50 years and most are female^19^^,^^24^.

VR approaches exclusively based on the biomedical model have been widely criticized and their effectiveness have been put into question especially when it comes to cost-benefits. More promising approaches and with more substantial impact should also consider sustainable return to work, focus on the worker’s satisfaction and engage coworkers, supervisors and employers^25^^,^^26^^,^^27^. Moreover, It is believed that the assessment of functioning and disability among people with RSI/WRMSDs on sick leave or not, should include their own perception of illness and demands to return to or remain at work, is an essential component in VR^6^^,^^11^. Studies in which the ICF Checklist has been adopted to analyze RSI/WRMSDs and other health problems point to the potential of this tool^14^^,^^15^^,^^16^^,^^23^. The results of the present study support the use of ICF by VR programs in two respects: first, as a possible way to explore the process of illness and disability by focusing on the impact of the illness on the individual’s functions; the second concerns the contribution in the development of a systematic instrument to measure the indirect consequences and social demands related to work disability as part of the its burden^28^^,^^29^.

Most codes in the RSI/WRMSDs Checklist within the domain activity/participation and environmental factors were linked to the participants’ narratives. Additional codes, i.e. not included in the RSI/WRMSDS Checklist but deserving attention for being frequently mentioned, should be considered in future validation studies. The interaction between domains activity/participation and environmental factors considered in the present study afforded a comprehensive understanding likely to improve the visibility of work-related aspects of disability.

The participants reported frequently difficulties in performing routine tasks. Many of these tasks were related to the category mobility, thus calling attention to the demands that affect everyday life activities. Some relevant examples are the codes d430 (lifting and carrying objects), d440 (precise hand movements ) and d445 (hand and arm use). These mobility issues can impact other activities, such as undertaking other manual tasks, carrying out daily routines and housework, as previously considered in another study^30^.

Doing housework (code d640) comprises many tasks, such as washing and drying clothes and garments, cleaning the kitchen and the utensils, cleaning the house, using household appliances and disposing garbage^1^. These housework tasks, as pointed out by Neves and Nunes^31^, reinforce the burden on full-time employed women, whom must complete manual tasks both at work and at home.

Another important code that immerged from this study’s data was d850 (remunerative employment) and it refers to workers’ difficulty in performing work tasks. As a rule, work incapacity happens when workers cannot continue to be productive , i.e. when workers cannot meet their production or job demands as a function of the physical limitations caused by disease^31^^,^^32^.

The participants also mentioned strategies and adjustments to complete routine tasks, such as taking breaks, performing tasks in several steps and replacing glassware and porcelain by plastic (due to light weight). In regards self-care, most participants reported to wear loose clothes and to keep their hair short; some reported making simple adjustments such as using hand pads to open doors. Some of these devices are represented in the code e115 (products and technology for personal use in daily living)^1^.

The efforts required to develop alternatives to minimize the demands related to mobility, personal care and housework was evident in the narratives and compelled the participants to reorganize or adjust their daily routine. It is an essential part of the VR to pay attention to these demands and to support the adaptations needed on these aspects of everyday life whenever possible^6^.

Most participants reported difficulty to perform and participate in leisure and recreation activities (code d920). According to the ICF, this code includes games, sports, arts and culture (going to a theater, museum or leisure readings), crafts and socialization, and other activities^1^. Participation in these activities among female workers with RSI/WRMSDs is significantly low as a function of their physical and/or emotional disabilities. This leads to a gradual social withdraw with negative impact on their social life, on their interpersonal relationships, mental health, quality of life and recovery^31^^,^^33^.

The participants reported to depend on support from their immediate and extended family and friends to accomplish daily tasks. This situation is represented by codes e310, e315, e320 and e325, all of which are included in the checklist. Social support is a significant facilitator when managing of RSI/WRMSDs. Indeed, social support is considered a facilitator, because it may minimize the individual incapacity to perform tasks and increase the affected person’s their confidence to perform activities and participate in social life. Individuals with high levels of social support are able to improve their physical and psychological performance, which might be associated with less functional disability and greater ability to face the barriers derived from the many aspects of the disease^34^^,^^35^^,^^36^.

Findings related to the use of medications (third level code e1101, re-categorized as the second level code e110) are contradictory; for some participants the use of medication could improve symptoms, especially those with chronic pain, while for others had a negative effect due to side effects, such as stomachache, excessive drowsiness and so forth^37^.

Most participants mentioned issues represented by codes e425, e430 and e450, as well as by codes e410, e415, e445, e455, e460 and e465. They are ll related to the attitudes of social groups based on different ideologies, values, rules and beliefs. These are attitudes that can influence individuals’ behavior and their social life and they involve aspects such as individual or social attitudes towards trustworthiness and the valorization of each human being, leading to either positive and inclusive or to negative and discriminatory practices^1^. Attitudinal barriers are one of the main challenges people with RSI/WRMSDs face because they are difficult to overcome. Social discrimination has significant impact on the VR process. These barriers derive from community-based opinions and coming from different social groups may lead to wrongly labelling individuals, allegedly revealing the failure of social systems to recognize illness and prevent work disability^31^^,^^32^.

The difficulty family members, coworkers and supervisors, and even healthcare providers have to acknowledge the legitimacy of disease and their lack of belief in its actual existence, triggers feelings of powerlessness among the affected individuals^25^. The same is the case of people with chronic pain and several other disorders^32^^,^^35^. Stigmatization and marginalization of individuals with RSI/WRMSDs is frequent in the world of work, because coworkers and supervisors often see them as a hindrance and become indifferent to their condition^25^^,^^26^^,^^27^.

One of the codes most frequently mentioned by the participants, e570, represents social security services, systems and policies. It refers to the financial support available when a worker needs to take sick leave while undergoing treatment and rehabilitation^1^. While this support might act as a facilitator since it affords conditions to complete treatment, it also can be a barrier to legally obtain temporary disability benefits while in sick leave. In Brazil, only insurance physicians affiliated with the social security administration may issue work disability certificates. Therefore, in addition to the difficulty to prove a causal link between disease and work, the affected workers must also deal with problems related to their right to sick pay, treatment and VR^5^^,^^6^.

Code e580 (health services, systems and policies) embodies health promotion, prevention, treatment and rehabilitation and includes a wide range of healthcare providers. This code, alluded in the participants’ narratives as need of medical care, treatment and adequate rehabilitation, can represent a facilitator or a barrier to access to healthcare services. The attitudes of health care providers, the quality of the treatment received, and the coordination of the entire care have different impact on the outcomes of treatment and VR^5^^,^^6^^,^^29^^,^^34^.

Labor and employment services, systems and policies (code e590) were significantly mentioned by the participants. This is a generic code that includes a wide variety of services and actions including workplace and employment services, e.g. job accommodations, vocational training and occupational health and safety programs, among others^1^. It is important to note that the ICF fails to identify a substantial part of demands related to the workplace system, including. some psychosocial and work-related demands and organizational policies that can impact on return to work, e.g. dismissal policy based on discrimination or voluntary resignation programs, lack of gradual return to work or modified duty options, etc^3^^,^^26^. Some of these issues were identified by participants as main obstacles to return to productive work. Therefore, a revision of the ICF environmental domain is suggested to include workplace demands associated with work disability, particularly ones related to improving workplace conditions required for a successful rehabilitation outcomes^25^^,^^27^. Finally, additional codes should be added and those that didn’t have a link to the participants’ narratives should be analyzed in future studies.

The main limitations of the present study derive from the sample characteristics. The convenience sampling comprised exclusively women. While RSI/WRMSDs affect mainly women, information on how both men and women describe these conditions and the specific rehabilitation demands should be considered in future studies.

## CONCLUSION

The present study reflects the efforts of a multiprofessional group to improve the utilization of an instrument to measure functioning and disability during the rehabilitation of workers with RSI/WRMSDs, from the workers’ perspective of the individual and environmental demands they experience. The approximation with the ICF vis-à-vis a qualitative research was beneficial to present an in-depth understanding of the evaluation and rehabilitation processes in the context of VR in Brazil. The analysis of workers’ narratives for the qualitative validation of the instrument, widen its scope and comprehension within a biopsychosocial framework.

Most codes included in the RSI/WRMSDs Checklist were confirmed by the participants’ narratives, while others not initially considered should be assessed for eventual addition to a revised version of this instrument. Fifty-three of the 60 included codes were confirmed, further 11 were added to domains activity/participation and environmental factors and 7 were excluded. These changes enable a better characterization of the limitations and demands experienced by female workers with RSI/WRMSDs in regard to their activities and social participation, as well as to environmental issues.

The utilization of the ICF and the RSI/WRMSDs Checklist might facilitate the detection of clinical and social problems experienced by workers during the return-to-work process (or staying at work process), not only related to the personal system, but also to workplace, compensation and healthcare systems. This perspective might contribute to the design of more effective public policies to deal with this complex problem. For this reason, we suggest future studies to also include men and the application of the checklist with representative samples of both male and female workers to attain other levels of validation.
